# Supplementary material for: Simulation Validation of an 8-Channel Parallel-Transmit Dipole Array on an Infant Phantom: Including RF Losses for Robust Correlation with Experimental Results
Source: Sensors (Basel). 2024 Apr 1;24(7):2254. doi: 10.3390/s24072254 (PMC11014297; doi:10.3390/s24072254)
Supplement: Supplementary file 1 [file sensors-24-02254-s001.zip › sensors-2898130-supplementary.pdf]

Supplementary Table S1 - Capacitor value (C2) for the built and simulated 8TxRx dipole coil array. Configurations 1 and 2 are given.

Capacitor C2 (pF)	Built coil	Configuration 1	Configuration 2
**Dipole 1**	18	18	18
**Dipole 2**	18	18	18.1
**Dipole 3**	20	25	24.9
**Dipole 4**	18	18.2	18.1
**Dipole 5**	20	24.9	25
**Dipole 6**	20	24.9	18
**Dipole 7**	20	22.6	18.1
**Dipole 8**	20	22.5	23.9

Supplementary Table S2 - Resistors values for the simulated 8TxRx dipole coil array in configuration 1

Resistors (Ohms)	R1	R2
**Dipole 1**	3	2.89
**Dipole 2**	2	2
**Dipole 3**	3	3
**Dipole 4**	0.20	1.06
**Dipole 5**	4.5	4.5
**Dipole 6**	0.63	2.89
**Dipole 7**	3.49	3.4
**Dipole 8**	3	2.99

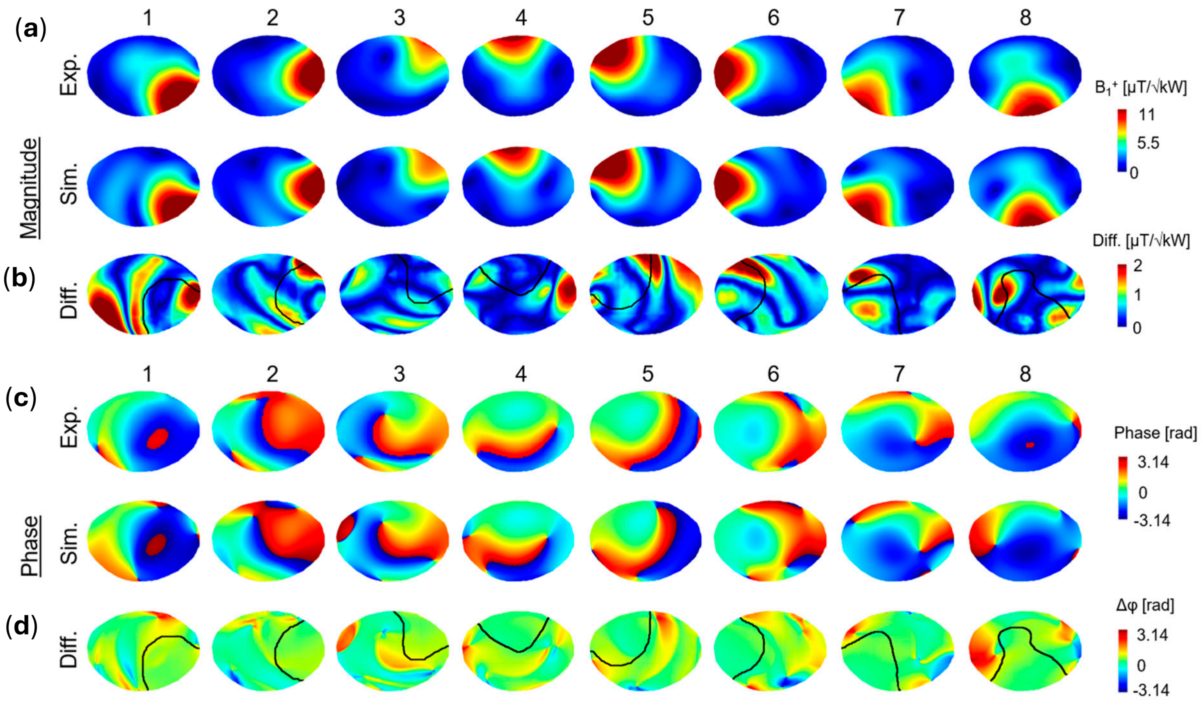

Supplementary Figure S1. In configuration 2 - (**a**) Experimental and simulated B_1^+-field maps, normalized to 1kW total input power, shown for individual dipoles. (**c**) Experimental and simulated individual phase maps, computed relative to the shimmed mode. (**b**) and (**d**) Difference maps calculated for the individual B_1^+-field maps with different scale than (**a**). In (**b**) and (**d**) a contour line was drawn (in black) to represent the experimental individual B_1^+-field distribution patterns.
